# Unveiling heterogeneous gaze patterns in autistic children undergoing rTMS: insights from latent profile analysis

**DOI:** 10.3389/fpsyt.2025.1665031

**Published:** 2026-01-12

**Authors:** Hong Liu, Chang Xu, Yingchen Xiao, Li Tian, Yin Li, Xin Zhang, Lei Gao

**Affiliations:** 1 Department of Mood Disorders II, Tianjin Anding Hospital, Tianjin, China; 2 Department of Editorial Office of Chinese Journal of Contemporary Neurology and Neurosurgery, Tianjin Huanhu Hospital, Tianjin, China; 3 Women’s Group Health Department, Tongzhou Maternal and Child Health Care Hospital of Beijing, Beijing, China; 4 Department of Health, Tongzhou Maternal and Child Health Care Hospital of Beijing, Beijing, China; 5 Diagnosis and Treatment Center for Neuromodulation, Tianjin Anding Hospital, Tianjin, China; 6 Medical School of Tianjin University, Tianjin University, Tianjin, China; 7 Department of Maternal, Child and Adolescent Health, School of Public Health, Tianjin Medical University, Tianjin, China

**Keywords:** autism spectrum disorder (ASD), repetitive transcranial magnetic, stimulation (rTMS), latent profile analysis (LPA), gaze patterns, tolerance

## Abstract

**Aim:**

Repetitive Transcranial Magnetic Stimulation (rTMS) is a promising intervention for Autism Spectrum Disorder (ASD), but objective markers for rTMS tolerance remain lacking. This case-control study explored gaze behavior variability toward emotional faces in ASD children undergoing rTMS, focusing on identifying subgroups associated with rTMS intolerance.

**Method:**

Eye-tracking data (Tobii technology, preferential-looking paradigm) were collected from 104 ASD children (48 intolerant, 56 tolerant) receiving DLPFC-targeted rTMS (left high-frequency, right low-frequency). Latent Profile Analysis (LPA) identified fixation subgroups across eight conditions. Demographic (sex, age), clinical (CARS scores) differences, and profile-tolerance associations were analyzed.

**Results:**

LPA revealed three gaze profiles: moderate non-preferential fixation (80.76%), low diverse fixation (9.62%), and increased fixation with mild variability (9.62%). Sex and CARS scores differed across profiles (age did not). Intolerance rates were 38.1%, 60.0%, and 100%, with profiles predicting rTMS intolerance (OR = 0.210, 95% CI: 0.079–0.557).

**Conclusion:**

ASD children exhibit heterogeneous emotional face gaze patterns. Findings highlight the need for personalized rTMS interventions, especially for those with higher CARS scores and increased emotional face fixation—at elevated intolerance risk.

Autism Spectrum Disorder (ASD) is a complex and severe neurodevelopmental disorder, core characteristics of which include persistent deficits in social communication and interaction, alongside restricted, repetitive patterns of behavior, interests, or activities (1). Over the past five decades, the global prevalence of ASD has shown a steady upward trend; in the United States alone, the prevalence reached 1 in 31 children by 2025 (2), highlighting the urgent need for effective clinical interventions and in-depth mechanistic research. A well-documented hallmark of ASD is impaired emotional processing, which manifests in multiple dimensions. Compared to typically developing children, individuals with ASD often struggle with recognizing the emotional states of others (3), exhibit reduced sensitivity to both positive and negative environmental cues (4), and face challenges in integrating social information, selecting social perspectives, and interpreting social cues—all of which further hinder their emotional understanding, expression, and social adaptation.

Notably, emotional recognition impairments in ASD are not limited to real-time social interactions but also extend to facial emotional recognition (FER) (5), a fundamental component of social cognition. However, existing research on FER in ASD has yielded inconsistent findings, reflecting substantial heterogeneity in outcomes (6, 7). This heterogeneity is attributed to multiple factors: on the one hand, methodological variations such as differences in stimulus characteristics (e.g., facial expression intensity, static *vs*. dynamic stimuli) and task designs (e.g., forced-choice *vs*. free-viewing paradigms) can significantly influence results (3, 6); on the other hand, intrinsic heterogeneity of ASD itself, including age, cognitive ability, and comorbidities, contributes to divergent FER performance (8). Given this complexity, characterizing distinct FER phenotypes is essential for refining diagnostic criteria, advancing etiological research, and developing targeted interventions—particularly in research and clinical contexts where precise subgroup identification can enhance the validity and reproducibility of findings.

Neuroimaging studies have identified a network of brain regions critical for FER, including the amygdala (central to emotional salience detection), basal ganglia (involved in reward processing and behavioral regulation), lateral and medial parietal cortex (supporting attention and social perspective-taking), and prefrontal cortex (mediating higher-order emotional regulation and executive control) (8, 9). In recent years, non-invasive brain stimulation (NIBS) techniques have emerged as promising tools to modulate neural activity in these regions, offering new avenues to improve FER and related social functions in both healthy populations and clinical groups, including individuals with ASD (10). Among NIBS approaches, repetitive transcranial magnetic stimulation (rTMS) has garnered increasing attention due to its ability to non-invasively modulate the excitability of specific brain regions and regulate functional connectivity within neural networks (11)—mechanisms that align with the neurophysiological underpinnings of ASD.

Early rTMS research in ASD primarily targeted the motor cortex, focusing on alleviating repetitive behaviors (12). However, as understanding of ASD pathophysiology has advanced, research has shifted toward brain regions more directly implicated in the disorder’s core symptoms (13). The dorsolateral prefrontal cortex (DLPFC) is one such key region: as a hub for higher-order cognitive functions, it plays a pivotal role in executive control, emotion regulation, and social information processing—domains that are consistently impaired in ASD (14). Neuroimaging evidence further supports that dysfunctional coordination between the DLPFC and the “social brain network” (including the amygdala, superior temporal sulcus, and precuneus) underlies the social interaction deficits and theory of mind impairments observed in ASD (15). Our previous work demonstrated that rTMS targeting the DLPFC improved gaze behavior toward emotional faces in individuals with ASD, suggesting potential benefits for FER and social attention (16).

Despite these promising findings, critical gaps remain in the field. First, while rTMS has shown preliminary efficacy for ASD-related symptoms, its generalizability is limited: some patients with ASD exhibit sensory hypersensitivity—a common comorbidity (17)—and may develop intolerance to rTMS coil stimulation, leading to treatment withdrawal. To date, no studies have explored whether FER impairment phenotypes can predict such treatment intolerance, nor have biomarkers for rTMS response in ASD been identified. Second, the long-term efficacy and sustainability of rTMS for ASD-related FER and social deficits remain understudied, with most existing research focusing on short-term outcomes (18). Third, the heterogeneity of FER impairment in ASD has not been adequately addressed using latent variable modeling—an approach that can identify non-arbitrary, homogeneous subgroups within heterogeneous clinical populations (19, 20). To our knowledge, no prior studies have applied latent profile analysis (LPA) to characterize subgroups of ASD based on emotional face processing patterns.

To address these gaps, the present study adopted the preferential-looking paradigm established in our previous work (16, 21) to collect gaze data during emotional face viewing in individuals with ASD. We then used LPA to identify latent subgroups based on these gaze patterns and further examined the association between these subgroups and treatment withdrawal (due to rTMS intolerance) in our prior clinical trial. This research aims to: (1) deepen understanding of the neural and behavioral mechanisms underlying FER heterogeneity in ASD; (2) identify clinical subtypes of ASD that are most likely to tolerate and benefit from current rTMS protocols; and (3) optimize resource allocation in clinical settings by reducing non-responsive treatment attempts. Ultimately, this work may inform the development of personalized, targeted rTMS interventions for ASD.

## 1 Methodology

### 1.1 Participants

We mainly collected ASD cases in Tianjin Anding Hospital from October 2018 to October 2023. The criteria of dropout (intolerance) mainly included: ①intolerance to the 1st rTMS intervention, such as adverse reactions to coil contact (distress or resistance) or intolerable side effects (including headache, dizziness, worsened anxiety, or seizures) leading to participant withdrawal or researcher-mandated discontinuation;② participants who have completed the first intervention but failed to the whole course (10 rTMS interventions). The eligibility criteria for dropout included: (1) it meets the diagnostic criteria of ASD in the fifth edition of the American Diagnostic and Statistical Manual of Mental Disorders (DSM-V); (2) age of 3–18 years old; (3) no medication during the rTMS intervention; (4) right-handed; (5) the total score of Childhood Autism Rating Scale (CARS) in the baseline ≥ 30 (22); and (6) less than 10 rTMS interventions. Similarly, ASD children who completed the whole rTMS intervention were selected as the control group with the same eligibility criteria except “complement at least two courses of rTMS intervention”, by matching chronological age, gender and socioeconomic status (SES). The exclusion criteria for both case and control group were: (1) contraindications to rTMS, such as metal or electronic instruments near the coil stimulation site; participants with a history of epilepsy (excluding epilepsy according to their electroencephalogram and medical record); participants with a history of brain trauma, brain tumors, and other diseases; participants with severe or recent heart disease; or other major physical illness. (2) Diagnosis of other mental illnesses (e.g., attention-deficit hyperactivity disorder, schizophrenia and depression). (3) Other neurodevelopmental disorders, genetic metabolic disease, or severe neurological disease. (4) Participants who could not cooperate with the eye movement experiment. (5)Participants who withdrew for non-medical reasons, such as scheduling conflicts or relocation, were not classified as intolerant. The study was conducted under the Code of Ethics of the World Medical Association (Declaration of Helsinki). Also, the study complied with all relevant national regulations and institutional policies and had been approved by the Medical Ethics Committee of Tianjin Medical University. Participants and their parents (or legal guardians) obtained all information about the research, including the purpose, requirements, responsibilities, compensation, risks, benefits, and alternatives. All questions were answered before asking for the consent signature.

### 1.2 Questionnaire and clinical assessments

We have used the self-made dropout questionnaire to investigate the parents of the dropout ASD patients through telephone or WeChat. The main contents included: demographic information of patients, clinical characteristics (such as the score of CARS, and IQ), reasons for withdrawal from treatment (Multiple-choice questions), the most intolerable thing for intervention (Single-choice Questions), and their feelings about rTMS intervention.

We evaluated the symptoms of ASD with CARS. The CARS consists of 14 domains assessing behaviors associated with autism, with a 15th domain “*rating general impressions”* of autism. Each field has a scale of one to four. Higher scores indicate a higher level of impairment. Total scores can range from 15 to 60. Scores below 30 mean that the individual is in the non-autistic range, a score between 30 and 36.5 indicates mild to moderate autism, and scores between 37 and 60 indicate severe autism.

### 1.3 Eye tracking procedure

**Figure 1:**
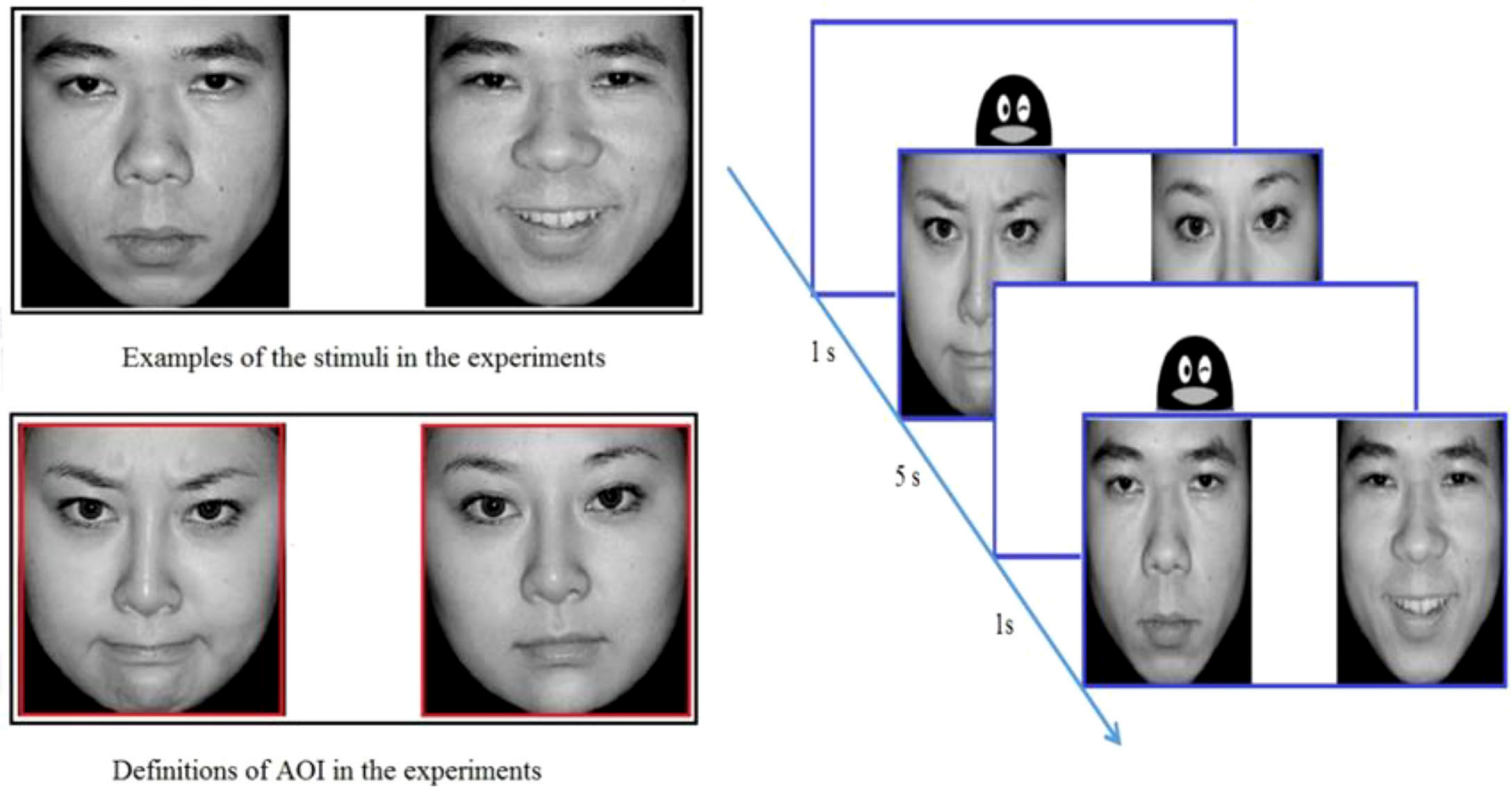
The examples, definitions of AOI & flowchart in the visual preference experiments.

The stimuli were selected from the Chinese Affective Picture System (23) (CAPS) and consisted of 48 different pictures (24 male faces and 24 female faces). Each picture included two black-and-white photographs of the same type portraying varied emotional valence (positive/negative + neutral) of the same person. Each picture was equal in size and symmetrical in position. There were three factors in this study, including the picture type (male, female), the left or right visual field where the emotional pictures presented (LVF, RVF), and the picture valence (positive, negative). There was one block for each condition and 6 trials in each block. Thus, 48 trials were included in total during the experiment. Examples of face stimuli are presented in **Figure 1**.

We used a Tobii TX300 eye tracker and the Tobii Studio software to present the stimuli, record eye movements, and analyze the gazing behavior of the participants. **Figure 1** showed the stimuli used in the experiments. The fixation was defined as continuous gazing for more than 80 ms within a 1 degree of visual angle or 30 pixels. The experiment took place in a controlled environment (illumination, temperature, etc.) in the development laboratory of the Department of Maternal, Child & Adolescent Health at Tianjin Medical University.

Participants were instructed to look at the pictures on the monitor in a relaxed way. After completing the 9-point calibration, the test started with instruction text displayed on the screen explaining the procedure in detail. The pictures were presented in randomized order for duration of 5 s at a sampling rate of 120 Hz by using Tobii Studio 3.0 Eye Tracking Software. Each picture had a size of 720×480 pixels, subtending a visual angle of 13.78° in height by 7in width. When appearing together, the pictures were located approximately 5°visual angle away from each other. Between two trials, an image of a cartoon penguin over a white background was presented at the center of the screen for 1 s. While viewing the picture, the subject was not required to give a response. For uncooperative subjects, the caregiver was allowed to accompany the participant, but not to see the screen. A flow chart of the experiment is presented in **Figure 1**.

We used the entire area occupied by the emotional face as the Area of Interest (AOI). We analyzed the total fixation duration (TFD), i.e. the sum of the duration of the subject’s fixation in the AOI (24). Percent of total fixation duration to emotional *vs*. neutral faces were calculated, i.e. sum of fixation time per AOI was divided by the total sum of fixation time for both AOIs to derive the proportion of time spent on each AOI (i.e., “% emotional” and “%neutral”) and to correct for missing data (25).

### 1.4 rTMS procedure

A trained electrophysiologist delivered rTMS stimulation over the cortical area controlling the contralateral First Dorsal Interosseous (FDI) using a Magnetic Field Stimulator (CCY-1, YIRUIDE Medical Corporation, Wuhan, China) to detect resting motor threshold (MT). The MT was determined for each hemisphere in all individuals by gradually increasing the output of the machine by 5% until a 5 mV deflection or a visible twitch in the FDI muscle was identified in 2 out of 3 trials (26). Electromyographic (EMG) responses were monitored continuously from the hand contralateral to the stimulated hemisphere using the MEP module in Magnetic Stimulator (YIRUIDE Medical Corporation, Wuhan, China). Subjects were familiarized with the laboratory and procedure before the first TMS session. In this study, rTMS was selected to stimulate left DLPFC with high frequency (10 Hz) and right DLPFC with low frequency (1 Hz) based on the evidence-based basis proposed by the European Union of Neurological Societies (27), and the electrode positioning cap was used for accurate positioning. Specific parameters are as follows: stimulation frequency of right dorsolateral prefrontal lobe is 1Hz, stimulation time is 32s, stimulation number is 32, intermittent time is 1s, repetition number is 28, the stimulation intensity is 25% MT; stimulation frequency of left dorsolateral prefrontal lobe is 10Hz, stimulation time is 3.2s, stimulation number is 32. Intermittent time is 10s, repetition number is 45. Stimulation intensity is 25% MT.

### 1.5 Latent profile analysis model

LPA aims to identify types of people with different personal and/or environmental attributes profiles. In the domain of vocational behavior, frequently these personal and environmental attributes are psychological constructs (e.g. different types of commitment, different dimensions of career adaptability, and different types of perceived environmental support), so LPA can also be described as identifying construct-based profiles (28). We introduce a common LPA model equations as shown below:


$$\sigma_{i}^{2}=\sum_{k=1}^{K}\pi_{k}{\left(\mu_{ik}-\mu_{i}\right)}^{2}+\sum_{k=1}^{K}\pi_{k}\sigma_{ik}^{2}$$

where μ_ik_ and σ_ik_ represent profile-specific (*k*) means and variances for variable *i*, and *π_k_* indicates profile density, or the proportion of N participants that belong to profile *k*. LPAs assume (a) samples drawn from a heterogeneous population produce data that are a mixture of *K* profile-specific distributions; (b) observed y indicator variables are distributed normally; and (c) the profile-specific mean vectors μ_k_ are the profile-specific (k) observed variable means.

### 1.6 Data analysis

LPA was conducted to investigate the optimal number of latent profiles that describe the patients’ fixation (mean TFD) on each of conditions of emotional faces. There were altogether 8 conditions, those were gender (male/female) × location(left/right visual field) ×valence (positive/negative). Models with two to seven profiles were tested. All analyses were conducted in Mplus 8.3 using robust maximum likelihood estimation. We used the following fit indicators to determine the optimal number of latent profiles (29, 30): Akaike information criterion (AIC), Bayesian information criterion (BIC), and adjusted Bayesian information criterion (aBIC) were employed to compare the model, with lower AIC, BIC, and aBIC values indicating a better model fit; the Lo-Mendell-Rubin (LMR) and bootstrap likelihood ratio test (BLRT) were examined to identify whether a k-class model fit better than a model with k-1 classes, and a significant p-value indicated that the k class was better; the *entropy* was assessed to identify each model’s classification precision with greater values implying more precise categorization (ideally above 0.80).

A regression mixture model (RMM) was used to analyze and compare the sex distribution, age, and group of individuals within the different profiles.

## 2 Results

### 2.1 Descriptive statistics

The dropout group consisted of fifty-six ASD participants, but five of them did not complete the eye-tracking experiments and three of them failed to finish the dropout questionnaire, and 48 remained at last, with a mean age of 7.995 (SD = 1.899; a range of 3.0–15.2) years. There were fifty-six ASD participants in the control group, with an average age of 8.802 (SD = 2.711; range of 3.3–17.8) years. There was no statistical difference (*t* = 1.080, *P* = 0.284) between the two groups. The sex distribution between the two groups was not statistically different either ( $\chi^{2}$=0.085, *P* = 0.771). Neither did the total score of CARS (38.96 ± 7.67 *vs*. 36.95 ± 6.82, *t* = 1.141, *P* = 0.258). see **Table 1**.

**Table 1:** The demographic information of the dropout group and control group.

Variables	Dropout group	Control group	t/χ^2^	*P*
N	48	56		
Age	7.995 ± 1.899	8.802 ± 2.711	1.080	0.284
Gender			0.085	0.771
male	38	43		
female	10	13		
Total CARS	38.96 ± 7.67	36.95 ± 6.82	1.141	0.258

### 2.2 Model fitness of LPA

**Figure 2:**
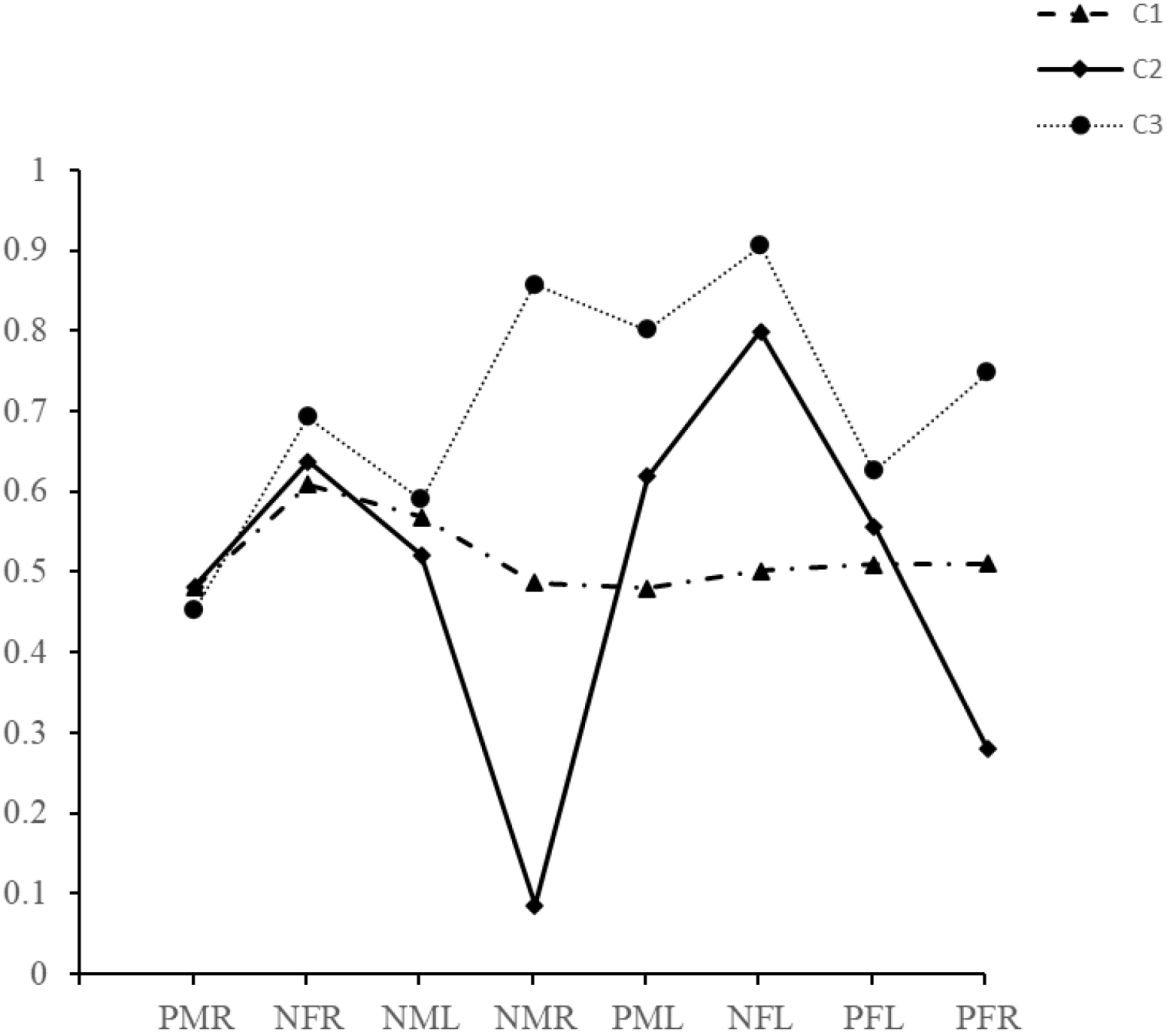
Latent profile indicators mean values for the three-profile solution. PMR, positive male face in RVF; NFR, negative female face in RVF; NML, negative male face in LVF; NMR, negative female face in RVF; PML, positive male face in LVF; NFL, negative female face in LVF; PFL, positive female face in LVF; PFR, positivefemale face in RVF.

As shown in **Table 2**, the AIC, and aBIC generally decreased as the number of estimated profiles increased and reached the least values at Model 6, while the BIC stopped decreasing at Model 3. As to entropy, it stayed consistently above 0.80. According to LMR, the four-class solution did not enhance model fit considerably compared with the three-class solution (*P* = 0.4585). However, BLRT indicated that the six-class solution was no better than the five-class solution (*P* = 0.999). Based on model fit tests and the goal of parsimony, the three-class solution was identified as the best description of latent facial fixation profiles. The latent profile memberships showed significant differences in the means of the eight indicator variables (**Table 3**), and their characteristics are summarized in **Figure 2**. Class 1 (n = 84, 80.76%), the moderate undeferential fixation group, was characterized by the average fixations on each condition without difference. Class 2 (n = 10, 9.62%), the less but diverse fixation group, was distinguished by a relatively less fixation but with intensive variance. Class 3 (n = 10, 9.62%), the more fixation with mild change group, was characterized by the highest fixation on emotional faces in most conditions.

**Table 2:** Fit statistics for the latent profile analysis.

Model	AIC	BIC	aBIC	ssaBIC	Entropy	LMR p-value	BLRT p-value	M%
2	1297.211	1371.106	-454.909	1292.005	0.911	0.0020	< 0.001	24.220
3	1206.686	1307.184	-508.771	1199.605	0.970	0.4388	< 0.001	9.550
4	1136.521	1263.622	-534.252	1127.567	0.909	0.5741	< 0.001	4.225
5	1074.987	1228.690	-542.808	1064.158	0.925	0.3982	< 0.001	4.225
6	1049.760	1230.065	-554.017	1037.057	0.908	0.9460	0.0000	4.225

AIC, Akaike information criterion; BIC, Bayesian information criterion; aBIC, adjusted Bayesian information criterion; LMR, the Lo-Mendell-Rubin test; BLRT, the bootstrap likelihood ratio test; M% refers to the proportion of the smallest category.

**Table 3:** Descriptive statistics for indicator variables that constituted the three profiles.

Variable	Class 1: moderate undeferential fixation group M (SD)	Class 2: less but diverse fixation group M (SD)	Class 3: more fixation with mild change group M (SD)	χ^2 #^	*P*
PMR	0.483 (0.184)	0.484 (0.071)	0.454 (0.308)	0.411	0.814
NFR	0.611 (0.140)	0.636 (0.209)	0.700 (0.143)	2.658	0.265
NML	0.570 (0.139)	0.522 (0.106)	0.592 (0.182)	0.506	0.777
NMR	0.490 (0.120)	0.086 (0.080)	0.862 (0.088)	48.334**	<0.001
PML	0.482 (0.195)	0.620 (0.162)	0.806 (0.139)	21.314*	<0.001
NFL	0.503 (0.138)	0.802 (0.181)	0.912 (0.084)	40.817*	<0.001
PFL	0.510 (0.209)	0.564 (0.275)	0.630 (0.028)	5.553*	0.062
PFR	0.512 (0.185)	0.284 (0.239)	0.750 (0.249)	14.731**	0.001

# We used the Kruskal-Wallis test due to heterogeneity of the variance. ** indicated there was statistical significance among the three groups, Class3>Class2>Class1. * indicated there was statistical significance between two groups, for PML, Class3>Class2& Class3>Class1; for NFL, Class3>Class1 & Class3>Class1; for PFL, Class3>Class1.

In addition, there was no statistical difference among the three latent profiles in terms of age, but not for sex distribution. However, the total score of CARS was statistically significant, but only between the “moderate undeferential fixation group” and “more fixation with mild change group”, the total CARS scores were significantly higher in the “moderate undeferential fixation group”, see **Table 4**. *LPA as a predictor of dropout*

**Table 4:** Descriptive statistics in the full sample and each latent profile.

Variable	Moderate undeferential fixation group	Less but diverse fixation group	More with mild change group	*F/χ^2^*	*P*
N	84	10	10		
Age	8.23 ± 3.57	8.10 ± 1.20	7.98 ± 1.78	0.030	0.971
Sex				6.621	0.036
male	65 (77.38%)	10 (100.00%)	6 (60.00%)		
female	19 (22.62%)	0 (0)	4 (40.00%)		
CARS*	41.29 ± 7.01	37.20 ± 4.39	34.70 ± 1.76	15. 584	<0.001
Dropout					
yes	32 (38.10%)	6 (60.00%)	10 (100.00%)	18.458	<0.001
no	52 (061.90%)	4 (40.00%)	0 (0)		

*Due to heterogeneity of variance, a Kruskal-Wallis test was used to compare the total score of CARS.

The RMM revealed significant differences between the three profiles regarding the group (χ^2^ = 18.458, *P* <0.001), but only between the “moderate undeferential fixation group” and “more with mild change group”. The intolerance rate in the three profiles was 38.1%, 60.0%, and 100%.And the latent profile was the influential factor of dropout, with β = -1.559,*S.E* = 0.497, *Wald* = 9.830, *P* = 0.002, *OR* = 0.210 (*95%CI*: 0.079, 0.557), which indicated that the participants in “less but diverse fixation group” or “more fixation with mild change group” are more easily to drop out than those in “moderate undeferential fixation group”.

## 3 Discussion

Latent Profile Analysis (LPA) was used to subgroup children with Autism Spectrum Disorder (ASD) based on their gaze behavior toward emotional faces, identifying three distinct profiles: moderate undeferential fixation group, less but diverse fixation group, and more fixation with mild change group. The largest proportion of patients belonged to the moderate undeferential fixation group, and they seemed to treat different emotional faces (with different genders, valence and in different visual fields) equally. This phenomenon indicated that they either lack attention to emotional faces or have an impairment in FER, unable to discriminate emotional faces. Additionally, patients in this group were more severe (with higher CARS scores) than those in “more fixation with mild change group”. This positive correlation between the severity of autism and FER impairment has been demonstrated in previous studies (31, 32). In contrast, patients in the remaining two latent profiles are minorities, with equal proportions, but they reflect differences in gaze for emotional faces in different situations. The second latent profile showed the most significant reduction in gaze for both male negative faces and female positive faces in the RVF, suggesting that the abnormal facial gazing behavior in this latent profile is partial rather than general. Interestingly, the ASD patients in this group, all of whom were male, showed the shortest gaze duration for the negative male faces in RVF, which could possibly be related to the avoidance of angry faces reported in previous studies (33, 34). In terms of “more fixation with mild change group”, they showed the most gazing at the emotional faces under most of the conditions, which indicated that their attention to emotional faces remained relatively intact.

More interestingly, the “more fixation with mild change group” completely failed to the rTMS treatment, which suggested the specialty of the phenotype, less severe (low score of CARS), more female (40%) and more fixation at emotional faces. We speculated that the possible reason for shedding in people with these characteristics may be related to anxiety. Because: (1) Females with ASD show greater internalizing symptoms (such as anxiety) than boys (35). In the phenotype, the proportion of female ASD was the highest. (2) Individuals with higher levels of anxiety are more likely to show increased gaze for emotional faces (36). In the latent profile, the participants showed the most gaze for emotional faces. And (3) anxiety disorders and sensory over-responsivity (SOR) are common in children with autism spectrum disorders (ASD), and there is evidence for an association between these two conditions (37). Even, anxiety was taken as a cause of SOR, according to the Primary Anxiety Model (38, 39). Meantime, SOR is more likely to become a possible reason for rTMS treatment intolerance. As we know, 56–79% of individuals with ASD (40, 41)showed the symptoms of SOR, which has been manifested as extreme sensitivity to stimuli such as unexpected loud noises or being touched (42). However, the interrelationship between anxiety and SOR is still controversial, for example, some researchers believe that anxiety and SOR are not causally related per se, but both are associated with abnormal activation of the amygdala (38, 43). In particular, for ASD individuals, evidence showed that amygdala size is positively correlated with anxiety and severity of social-communication symptoms compared to typically developing children (44, 45). Further, there has already been research on modulating amygdala activity through a ventrolateral prefrontal cortex (vlPFC) -amygdala white matter pathway by TMS. Our findings not only characterize the latent profile of ASD children who are intolerant of current TMS protocols, but also provide possible alternatives (such as vlPFC).

There are some limitations to this study. Firstly, the sample size is relatively small, although the sample size assessed by PASS is sufficient. We only verified the tolerance of a single protocol of rTMS, but our previous studies have confirmed that the current protocol is much more tolerant than others. Also, we could not further explore the neural substrates of the intolerance of rTMS. We will combine functional near-infrared spectroscopy (fNIRS) or fMRI to probe the mechanisms of rTMS interventions for intolerant individuals and select the optimal rTMS protocol in the ASD population.

## Data Availability

The raw data supporting the conclusions of this article will be made available by the authors, without undue reservation.
